# Protein Structure-Function Relationship at Work: Learning from Myopathy Mutations of the Slow Skeletal Muscle Isoform of Troponin T

**DOI:** 10.3389/fphys.2016.00449

**Published:** 2016-10-13

**Authors:** Anupom Mondal, J.-P. Jin

**Affiliations:** Department of Physiology, Wayne State University School of MedicineDetroit, MI, USA

**Keywords:** troponin isoform, skeletal muscle, slow twitch fiber, *TNNT1* myopathies, recessive mutation

## Abstract

Troponin T (TnT) is the sarcomeric thin filament anchoring subunit of the troponin complex in striated muscles. A nonsense mutation in exon 11 of the slow skeletal muscle isoform of TnT (ssTnT) gene (*TNNT1*) was found in the Amish populations in Pennsylvania and Ohio. This single nucleotide substitution causes a truncation of the ssTnT protein at Glu^180^ and the loss of the C-terminal tropomyosin (Tm)-binding site 2. As a consequence, it abolishes the myofilament integration of ssTnT and the loss of function causes an autosomal recessive nemaline myopathy (NM). More *TNNT1* mutations have recently been reported in non-Amish ethnic groups with similar recessive NM phenotypes. A nonsense mutation in exon 9 truncates ssTnT at Ser^108^, deleting Tm-binding site 2 and a part of the middle region Tm-binding site 1. Two splicing site mutations result in truncation of ssTnT at Leu^203^ or deletion of the exon 14-encoded C-terminal end segment. Another splicing mutation causes an internal deletion of the 39 amino acids encoded by exon 8, partially damaging Tm-binding site 1. The three splicing mutations of *TNNT1* all preserve the high affinity Tm-binding site 2 but still present recessive NM phenotypes. The molecular mechanisms for these mutations to cause myopathy provide interesting models to study and understand the structure-function relationship of TnT. This focused review summarizes the current knowledge of TnT isoform regulation, structure-function relationship of TnT and how various ssTnT mutations cause recessive NM, in order to promote in depth studies for further understanding the pathogenesis and pathophysiology of *TNNT1* myopathies toward the development of effective treatments.

## Troponin in vertebrate striated muscles and fiber type-specific isoforms

Vertebrates have two types of striated muscles, i.e., skeletal muscle and cardiac muscle. The basic contractile apparatus of vertebrate striated muscle is the sarcomeres that are in tandem repeats in the myofibrils. The sarcomeres consist of overlapping myosin thick filaments and actin thin myofilaments (Tobacman, 1996; Gordon et al., 2000). Muscle contraction is powered by actin-activated myosin ATPase (Cooke, 1986), which is regulated by intracellular Ca^2+^ through the troponin complex associated with the thin filament (Gordon et al., 2000). The binding of Ca^2+^ to troponin induces a series of allosteric changes in the thin filament, allowing the myosin head to form a strong cross-bridge with F-actin to activate myosin ATPase and initiate contraction (Leavis and Gergely, 1984).

Vertebrate skeletal muscle contains slow twitch and fast twitch types of fibers (Eddinger et al., 1985; Sosnicki et al., 1989). Correspondingly, muscle myosin and troponin have both evolved into slow and fast fiber type-specific isoforms. Slow and fast skeletal muscle fibers express type I and type II myosin, respectively, and these myosin isoenzymes differ in their ATPase activity (Bárány, 1967). Previous studies in multiple laboratories have demonstrated the contribution of four skeletal muscle myosin heavy chain (MHC) isoforms (type I, IIa, IIb, and IIx) to the magnitude and velocity of contraction of different types of muscle fibers (Ruff and Whittlesey, 1991; Johnson et al., 1994).

The troponin complex is at the center of the Ca^2+^-regulation of muscle contraction (Leavis and Gergely, 1984). Troponin consists of three protein subunits: The Ca^2+^-binding subunit TnC, the inhibitory subunit TnI, and the tropomyosin-binding subunit TnT (Greaser and Gergely, 1973). To convert the cellular signal of rising cytosolic Ca^2+^ originated from sarcolemmal electrical activity to myofilament movements, troponin functions through cooperative interactions among the three subunits and with tropomyosin and the actin thin filament (Tobacman, 1996; Gordon et al., 2000).

Among the three subunits of troponin, TnC belongs to a family of Ca^2+^ signaling proteins including calmodulin and myosin light chains (Collins, 1991). A fast isoform of TnC is found in fast twitch skeletal muscle fibers (Gahlmann and Kedes, 1990), whereas slow twitch skeletal muscle and cardiac muscle share another isoform of TnC (Parmacek and Leiden, 1989). In contrast, TnI and TnT are striated muscle-specific proteins, and each has diverged into three homologous isoforms corresponding to the cardiac, slow skeletal and fast skeletal types of muscle fibers (Hastings, 1997; Perry, 1998).

The three TnI and three TnT isoform genes are closely linked in three pairs in the chromosomal genome of vertebrates. The fast TnI and fast TnT genes are linked in one pair (Barton et al., 1997), which is consistent with their linked functions in adult skeletal muscles. However, the cardiac TnI gene is linked to the slow TnT gene (Huang and Jin, 1999) and the slow TnI gene is linked to the cardiac TnT gene (Tiso et al., 1997), which are different from their linked fiber type-specific expressions in adult slow skeletal muscle or heart. While such scrambled linkages of the two pairs of TnI and TnT genes indicate that the TnT and TnI isoform gene expression is regulated by the cellular environment rather than by genomic organization, slow TnI and cardiac TnT express and function together in embryonic heart (Jin, 1996). Embryonic cardiac muscle expresses solely slow skeletal muscle TnI that is replaced by cardiac TnI during late embryonic and early postnatal development (Saggin et al., 1989; Jin, 1996). Therefore, they are originally a functional pair of linked genes, whereas the cardiac TnI and slow TnT genes emerged later as the newest pair (Chong and Jin, 2009).

Mutations in the three TnT isoform genes *TNNT1, TNNT2*, and *TNNT3* encoding slow skeletal muscle TnT, cardiac TnT, and fast skeletal muscle TnT, respectively, have been reported to cause cardiac and skeletal myopathies. During the last two decades, numerous mutations in *TNNT2* gene have been found to cause various types of cardiomyopathies (Knollmann and Potter, 2001; Sheng and Jin, 2014). In contrast, rather few mutations in skeletal muscle TnT isoform genes have been reported in skeletal muscle diseases (Wei and Jin, 2016), among which five mutations in the *TNNT1* gene encoding slow TnT cause nemaline myopathies (NM). A nonsense mutation in *TNNT1* was first identified in the Old Order Amish (Johnston et al., 2000). The identification and mechanistic studies of the Amish NM (ANM) (Jin et al., 2003; Wang et al., 2005) have raised clinical awareness and the inclusion of testing for *TNNT1* mutations in the diagnosis of myopathies. As a result, four more truncation or internal deletion mutations in *TNNT1* gene have recently been reported in multiple other ethnic groups around the world to cause myopathies similar to that of ANM (van der Pol et al., 2014; Marra et al., 2015; Abdulhaq et al., 2016). No effective treatment is currently available for *TNNT1* NM.

Nemaline myopathies are neuromuscular disorders characterized by muscle weakness and rod-shaped or “nemaline” inclusions in skeletal muscle fibers (Wallgren-Pettersson et al., 2011; Nance et al., 2012). The different *TNNT1* NM mutations all have recessively inherited lethal phenotypes, indicating that the various truncations or internal deletion of slow TnT all result in the loss of function. This notion and the critical importance of slow skeletal muscle fibers have been confirmed for the case of ANM mutation (Jin et al., 2003; Wang et al., 2005). To understand the molecular basis for the pathogenesis and pathophysiology of *TNNT1* NM will provide insights into the structure-function relationship of TnT and the mechanism of muscle contraction. The present review is thus focusing on the myopathy mutations of slow skeletal muscle isoform of TnT. It is our hope that the in depth discussions will help to stimulate further research leading to the development of targeted treatment of these lethal skeletal muscle diseases.

## Structure-function relationship of troponin T

TnT is a 30–35-kDa protein (Greaser and Gergely, 1973). Based on available sequence information, the length of vertebrate TnT polypeptide chain ranges from 223 to 305 amino acids. This large size variation of TnT isoforms across species is almost entirely due to the variable length of the N-terminal variable region, from nearly absent in some fish fast skeletal muscle TnT to more than 70 amino acids long in avian and mammalian cardiac TnT (Jin et al., 2008; Wei and Jin, 2011; Jin, 2016). Primary structural data showed that while the N-terminal region is hypervariable in length and amino acid sequences, the amino acid sequences of the middle and C-terminal regions of TnT are highly conserved among the three muscle-type isoforms and across vertebrate species (Jin et al., 2008; Jin, 2016; Wei and Jin, 2016).

Electron microscopic studies showed that the TnT molecule has an extended conformation (Cabral-Lilly et al., 1997; Wendt et al., 1997). High-resolution X-ray crystallographic structures have been obtained for the core region of human cardiac troponin complex (Takeda et al., 2003) and chicken fast skeletal muscle troponin complex (Vinogradova et al., 2005). These solved high-resolution structures of the troponin complex contained entire TnC and most regions of TnI, but only a small C-terminal portion of TnT. The data showed that TnT interfaces with TnI in a coiled-coil structure (i.e., the I-T arm) formed by the segments of L^224^-V^274^ of cardiac TnT and F^90^-R^136^ of cardiac TnI in human cardiac troponin or E^199^-Q^245^ of fast TnT and G^55^-L^102^ of fast TnI in chicken fast skeletal muscle troponin. The C-terminal portion of the I-T arm also interacts with TnC. The observation that the N-terminal and the middle region as well as the very C-terminal end of TnT were not resolved in the high resolution crystallographic structures implicates a flexibility of these regions, likely reflecting their allosteric functions in the troponin regulation of muscle contraction.

**Figure 1:**
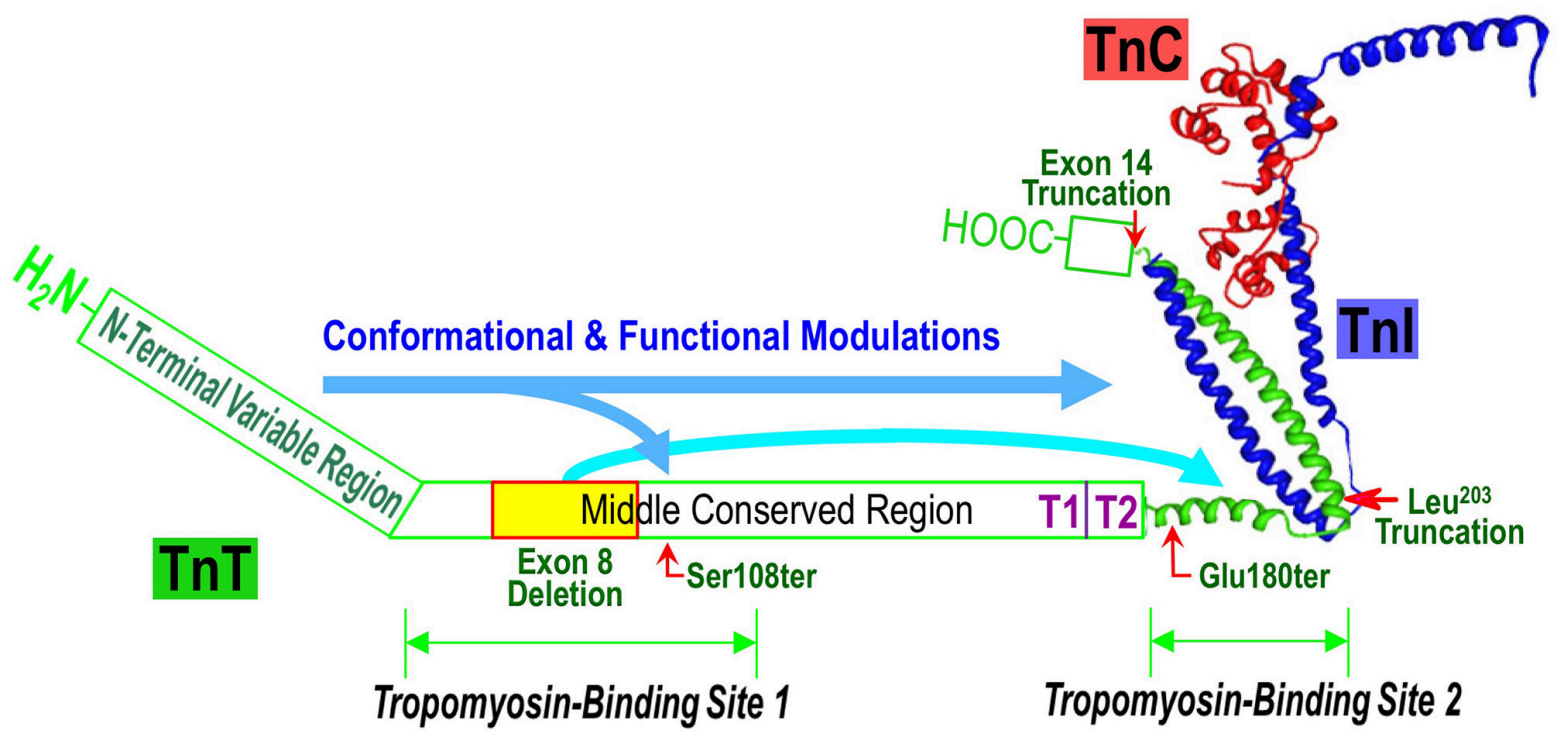
**Structural and functional domains of TnT and ***TNNT1*** NM mutations**. Illustrated on a structure-function model of the troponin complex (the crystallographic structure was adapted from Takeda et al., 2003), the positions of slow TnT Ser^108^, Glu^180^, Leu^203^ and exon 14 truncations and exon 8 deletion are indicated together with the interaction sites for tropomyosin (Tm), TnI and TnC. The long arrows propose the effects of N-terminal variable region on the overall conformation and function of TnT. In this model, the effect of N-terminal segment on decreasing Tm-binding affinity was augmented by the internal deletion of the exon 8-encoded segment.

Complementary to the crystallographic structure, the functional sites and the structure-function relationship of TnT have been extensively investigated in protein binding studies using TnT fragments generated from limited chymotryptic and CNBr digestions. The structural and functional domains of TnT are summarized in Figure 1. The classic chymotryptic fragments T1 and T2 of rabbit fast skeletal TnT (Tanokura et al., 1981) were studied for their bindings with other regulatory proteins in muscle thin filament. The ~100 amino acids C-terminal chymotryptic fragment T2 interacts with TnI and TnC and binds to the middle region of tropomyosin (Heeley et al., 1987; Schachat et al., 1995). The chymotryptic fragment T1 that contains both the N-terminal variable region and the middle conserved region of TnT binds the head-tail junction of tropomyosins in the actin thin filament (Heeley et al., 1987; Schachat et al., 1995). The tropomyosin-binding activity of the T1 fragment resides in the 81 amino acids CNBr fragment CB2 of rabbit fast skeletal muscle TnT, which represents a largely α-helical structure (Pearlstone et al., 1976, 1977) in the middle conserved region of TnT. The CNBr fragment CB3 of rabbit fast skeletal muscle TnT (amino acids 2-50) representing the N-terminal variable region does not bind TnI, TnC, or tropomyosin (Pearlstone and Smillie, 1982; Ohtsuki et al., 1984; Heeley et al., 1987; Perry, 1998).

More recent studies using genetically engineered TnT fragments and mapping with site-specific monoclonal antibody probes showed that the T1 region tropomyosin-binding site 1 of TnT included a 39-amino acid segment at the N-terminal portion of the middle conserved region of TnT (Jin and Chong, 2010). Its downstream boundary was further extended to beyond S^108^, the site of a *TNNT1* NM nonsense mutation that causes partial destruction of the tropomyosin binding site 1 (Amarasinghe et al., 2016). The tropomyosin binding site 2 in the T2 fragment was mapped to a 25-amino acid segment at the beginning of the T2 fragment (Jin and Chong, 2010). Amino acid sequences of these segments containing the two tropomyosin binding sites are highly conserved in the three muscle-type specific TnT isoforms and across vertebrate species (Jin et al., 2008).

Although, the N-terminal region of TnT does not bind any known myofilament proteins, its structure is regulated by alternative splicing during late embryonic and early postnatal development of the heart (Jin and Lin, 1988) and skeletal muscles (Wang and Jin, 1997), and in pathologic adaptation (Larsson et al., 2008). These developmental and adaptive regulations of the N-terminal variable region of TnT suggested functional significances. The entire N-terminal variable region of cardiac TnT can also be selectively removed during cardiac adaptation to acute energetic crisis by restrictive proteolysis (Zhang et al., 2006; Feng et al., 2008). Similar modification can also be produced in fast skeletal muscle TnT (Zhang et al., 2006).

*In vitro* studies have demonstrated the role of the N-terminal region in altering the molecular conformation of TnT in the middle and C-terminal regions and the interactions with TnI, TnC, and tropomyosin (Wang and Jin, 1998; Jin and Root, 2000; Jin et al., 2000; Biesiadecki et al., 2007). The physiological and pathological significances of the regulatory effects of the N- terminal variable region of TnT have also been demonstrated in *ex vivo* working heart and cardiomyocyte studies using transgenic mice expressing N-terminal modified TnT in the heart (Pan et al., 1991; Chandra et al., 1999; Biesiadecki et al., 2002; Feng et al., 2008; Wei et al., 2010; Wei and Jin, 2015).

Protein binding studies further demonstrated that the N-terminal variable region of TnT remotely modulates the binding affinity of TnT for tropomyosin by reducing the affinities of both site 1 in the middle region (Amarasinghe and Jin, 2015) and site 2 in the C-terminal region (Amarasinghe et al., 2016). This inhibitory regulation has been most clearly demonstrated in the case of the *TNNT1* exon 8 deletion NM mutant, where removal of the N-terminal segment very effectively restored tropomyosin binding affinity diminished by the mutation (Amarasinghe et al., 2016). These data further suggest that the conserved structures in the middle and C-terminal regions of TnT confer a baseline state of troponin function that is similar for cardiac, slow and fast skeletal muscle isoforms. The diverged N-terminal structure of the muscle type-specific TnT isoforms provides a regulatory mechanism to fine tune the function of troponin adapted to the contractility requirement in different muscle types and in physiological and pathophysiological adaptations.

The 9 amino acids at the very C-terminal end of TnT are highly conserved among the three muscle type isoforms and across vertebrate species (Jin et al., 2008). The functional significance of this segment has been an interest of experimental research. There is no direct evidence for binding of this C-terminal segment of TnT with any other myofilament proteins. This segment was not resolved in the high-resolution crystal structure of either cardiac or fast skeletal muscle troponin complex (Takeda et al., 2003; Vinogradova et al., 2005), implicating its possible nature as a flexible and allosteric structure. The recent finding of a splicing site mutation in *TNNT1* gene, which deletes the exon 14-encoded segment of the C-terminal 14 amino acids and causes NM (van der Pol et al., 2014), supports a critical role of the conserved C-terminal segment of TnT.

Consistently with this notion, mutations of single amino acid substitutions (R278C or R286C) in the C-terminal end segment, partial deletion (W287ter) or error-splice out of exon 17 encoding this segment in cardiac TnT have been found to cause cardiomyopathy (Thierfelder et al., 1994; Watkins et al., 1995; Richard et al., 2003). Biochemical and biophysical studies have demonstrated that the R278C mutant of cardiac TnT produces a slightly increased Ca^2+^ sensitivity with a significant elevation of sub-half-maximal force (Morimoto et al., 1999). Deletion of the C-terminal 14 amino acids of cardiac TnT resulted in lower level activation of myofilament ATPase with reduced effectiveness of Ca^2+^-troponin to switch the thin filament from the off to the on state (Mukherjea et al., 1999) and also caused detectable ATPase activation in the absence of Ca^2+^ showing hindered ability of regulated actin filament in conferring the inactive state (Franklin et al., 2012).

## Genes encoding troponin T isoforms

Three homologous genes have evolved in vertebrate species encoding the cardiac (*TNNT2*), slow skeletal muscle (*TNNT1*) and fast skeletal muscle (*TNNT3*) isoforms of TnT (Cooper and Ordahl, 1985; Breitbart and Nadal-Ginard, 1986; Jin et al., 1992; Farza et al., 1998; Huang et al., 1999; Hirao et al., 2004). It has been shown in avian and mammalian species that *TNNT1* and *TNNT3* genes specifically express in the slow and fast twitch skeletal muscle fibers, respectively. In contrast, *TNNT2* gene expresses in embryonic and adult cardiac muscle as well as transiently expresses in embryonic and neonatal skeletal muscles, including both slow and fast fiber dominant muscles (Toyota and Shimada, 1983; Cooper and Ordahl, 1985; Jin, 1996).

The functional diversity of TnT isoforms has physiological significances. An interesting example is that the cardiac muscle of toad (*Bufo*) expresses exclusively slow skeletal muscle TnT together with cardiac forms of TnI and myosin (Feng et al., 2012). This is a unique case since all vertebrate species studied to date from fish to human including the closely related genus frog (*Rana*) express only cardiac TnT in the cardiac muscle. Analysis of cardiac function demonstrated that toad hearts generated lower maximum stroke volume but significantly higher resistance to the increase of afterload than that of frog hearts (Feng et al., 2012). This feature is consistent with the unique functional requirement for the toad heart to work under drastically fluctuation of blood volumes and regulation via vasoconstrictions. This finding demonstrates a fitness selection value of the evolutionary adaptation of utilizing slow skeletal muscle TnT in toad cardiac muscle. The specific structure(s) of slow TnT in altering the contractility of toad cardiac muscle is worth investigating in order to better understand the critical role of slow TnT in skeletal muscle function as well as the development of a way targeting cardiac TnT to treat heart failure.

The evolutionary linage of the three TnT isoform genes have been thoroughly investigated by sequence analysis and protein epitope studies (Chong and Jin, 2009). Using monoclonal antibodies as site-specific epitope probes, a method was developed to detect evolutionarily suppressed molecular conformation by removing the suppressor structures, such as the evolutionarily added N-terminal variable region. The results demonstrated three-dimensional structure evidence for the evolutionary relationships between TnI and TnT and among their muscle type-specific isoforms (Chong and Jin, 2009). The data further demonstrate a novel mode of protein evolution by allosterically suppressing the ancestral molecular conformation with the evolutionary addition of a modulatory structure, in which the present-day form of TnT isoforms with diverged primary and folded structures have the potential of restoring ancestral conformations after removing the evolutionarily added repressor structure (Chong and Jin, 2009).

The adult heart and skeletal muscles express the three TnT isoform in a muscle fiber type-specific manner (Jin, 2016). Knockout of the *TNNT2* gene encoding cardiac TnT resulted in embryonic lethality (Nishii et al., 2008). Consistent with the differentiated role of slow muscle fibers critical to the mobility of animals (Rome et al., 1988), the loss of ssTnT results in severe NMs (Johnston et al., 2000; Jin et al., 2003; van der Pol et al., 2014; Marra et al., 2015; Abdulhaq et al., 2016). Therefore, the three muscle type TnT isoforms play non-redundantly roles in the functions of the three types of striated muscle.

In addition to the sequence and protein conformation lineage data, the undifferentiated utilization of the same TnC isoform in cardiac and slow skeletal muscles also supports the hypothesis that the emergence of the cardiac and slow TnI-TnT gene pairs was a relatively recent event of evolutionary divergence (Chong and Jin, 2009). A further support to this notion that among the three TnI-TnT gene pairs, cardiac TnI-slow TnT genes form the newest pair is the presence of a unique N-terminal extension in cardiac TnI, an additional structure that is absent in fast and slow skeletal muscle TnI isoforms (Parmacek and Solaro, 2004). The latest emergence of the slow TnT gene may be a landmark of vertebrate evolution and its functional significance requires more investigation.

## Alternative splicing

Expression of the three TnT isoform genes is regulated at the transcriptional level as well as via alternative RNA splicing (Jin et al., 2008; Wei and Jin, 2011, 2016). The splicing variants add to the diversity of TnT structure for fine tuning of muscle contractility during development and in adaptation to physiological stress and pathological conditions.

### Cardiac TnT

The mammalian cardiac TnT gene (*TNNT2*) contains 14 constitutively expressed exons and three alternatively spliced exons (Jin et al., 1992, 1996; Farza et al., 1998). Exon 5 of cardiac TnT gene, which encodes 9 or 10 amino acids in the N-terminal variable region, is included in embryonic but not adult cardiac TnT (Jin and Lin, 1989). Exon 4 of cardiac TnT gene is alternatively spliced independent of developmental state (Jin et al., 1996). The avian cardiac TnT gene contains 16 constitutively spliced exons and only one alternative exon (the embryonic exon 5) (Cooper and Ordahl, 1985). Correspondingly, four mammalian and two avian cardiac TnT N-terminal alternative splicing variants have been found in normal cardiac muscle.

The inclusion or exclusion of exon 5 generates an embryonic to adult cTnT isoform switching during development (Cooper and Ordahl, 1985; Jin and Lin, 1988; Jin et al., 1996). When *TNNT2* gene is transiently expressed in embryonic and neonatal skeletal muscles, the alternative splicing pattern is synchronized to that in the heart (Jin, 1996). The timing of the switching of *TNNT2* alternative splicing varies in different species, indicating regulation by a systemic clock, rather than adaptation to changes in contractile function (Jin, 1996).

Splice out of exon 4 that encodes 4-5 amino acids in the N-terminal variable region of cardiac TnT increases in failing human hearts (Anderson et al., 1995; Mesnard-Rouiller et al., 1997), diabetic (Akella et al., 1995) and hypertrophic (McConnell et al., 1998) rat hearts. Aberrant splice out of N-terminal coding exons of cardiac TnT (exon 7 in dogs equivalent to exon 8 in turkey) is found in dilated cardiomyopathy (Biesiadecki et al., 2002; Biesiadecki and Jin, 2002).

There is another alternatively spliced exon (exon 13) encoding a short segment of 2 or 3 amino acids between the T1 and T2 regions of mammalian cardiac TnT (Jin et al., 1992, 1996). The functional significance of this variable region is unknown.

### Fast skeletal muscle TnT

Mammalian fast skeletal muscle TnT gene contains 19 exons, of which exons 4, 5, 6, 7, 8, and a fetal exon encoding segments in the N-terminal variable region are alternatively spliced (Breitbart and Nadal-Ginard, 1986; Briggs and Schachat, 1993; Wang and Jin, 1997). Additional alternative N-terminal coding exons are present in avian *TNNT3* gene (Smillie et al., 1988; Ogut and Jin, 1998; Miyazaki et al., 1999; Jin and Samanez, 2001). Seven P exons are located between exon 5 and 6 in the N-terminal variable region of avian fsTnT encode a unique Tx segment (Smillie et al., 1988; Jin and Smillie, 1994; Miyazaki et al., 1999; Jin and Samanez, 2001). A w exon and a y exon are found between exons 4-5 and 7-8, respectively (Schachat et al., 1995).

The alternative splicing of two mutually exclusive C-terminal exons (16 and 17) each encoding a segment of 14 amino acids also occurs in *TNNT3* expression (Wang and Jin, 1997). This alternatively spliced segment of fast TnT is in the interface with TnI and TnC (Wei and Jin, 2016). Incorporation of exon 17-encoded segment weakened binding of TnT to TnC and tropomyosin (Wu et al., 1995). This region also shows diversity between mammalian and avian cardiac TnT, where the avian cardiac TnT gene contains an additional exon encoding two amino acids (Cooper and Ordahl, 1985).

Like that of cardiac TnT, expression of *TNNT3* gene undergoes a high to low molecular weight, acidic to basic isoelectric point splice form switch during development due to alternative inclusions of N-terminal exons (Jin et al., 2008; Wei et al., 2014). The alternative splicing of *TNNT3* pre-mRNA is regulated independently of skeletal muscle fiber types as deficiency of slow skeletal TnT did not affect the developmental switch of fast TnT splice forms (Wei et al., 2014).

### Slow skeletal muscle TnT

The slow skeletal muscle TnT gene *TNNT1* has a simpler structure and fewer alternative-splicing variants than that of the fast and cardiac TnT genes. There are only 14 exons in the *TNNT1* gene with one alternatively spliced. With exon-intron organizations same as that of the mammalian slow TnT gene (Huang and Jin, 1999), chicken slow TnT gene is significantly smaller (~3-kb versus ~11-kb) due to shorter introns (Hirao et al., 2004). Alternative splicing of exon 5 in the N-terminal region generates two variants of slow TnT (Gahlmann et al., 1987; Jin et al., 1998; Huang and Jin, 1999). Splicing at alternative acceptor sites in intron 5 of mouse slow TnT gene produces a single amino acid variation in the exon 6-encoded segment (Huang and Jin, 1999). The same pattern was found for the splicing of intron 4-exon 5 of chicken slow TnT gene (Hirao et al., 2004). Abnormal inclusion of 48 bases of the 3′-region of intron 11 was reported in a cloned human slow TnT cDNA (Gahlmann et al., 1987). However, no corresponding high molecular weight slow TnT protein was detectable (Jin et al., 1998), implicating a circumstantial splicing error.

Alternative splicing of slow TnT shows no apparent developmental regulation but may play a role in modulating muscle contractility in physiological and pathophysiological adaptations. While the high molecular weight splice form including the exon 5-encoded segment is the major slow TnT expressed in normal muscles, the low molecular weight slow TnT became predominant in overused prior polio muscle and significantly up-regulated in type 1 (demyelination), but not type 2, Charcot-Marie-Tooth disease (Larsson et al., 2008). Interestingly, the expression of slow skeletal muscle TnT in the toads hearts as an evolutionarily selected cardiac adaptation to the drastic changes in blood volume is solely the low molecular weight splice form (Feng et al., 2012). These observations indicate differentiated functionalities of the alternative spliced variants of slow TnT.

Aberrant splicing of slow TnT causes NM (van der Pol et al., 2014; Abdulhaq et al., 2016). Error splice out of the exon 8-encoded segment in the middle region of slow TnT drastically alters the molecular conformation and function in the C-terminal region and diminishes the binding affinity for tropomyosin (Amarasinghe et al., 2016).

## Nemaline myopathy mutations in *TNNT1* gene

**Figure 2:**
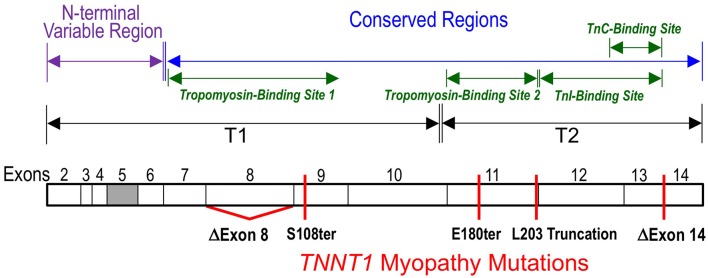
**Myopathic mutations of ***TNNT1*****. Locations of the nonsense and splicing mutations found in *TNNT1* gene that truncate the slow TnT polypeptide chain or causes an internal deletion are illustrated on a linear map of slow TnT protein with the segment encoded by exons 2-14 outlined. The filled box indicates alternatively spliced exon 5. All of the five *TNNT1* mutations cause recessively inherited nemaline myopathies. The known binding sites for TnI, TnC and tropomyosin are outlined.

Multiple mutations in *TNNT1* gene, located at 19q13.42 in the human genome, have been identified to cause autosomal recessively inherited nemaline myopathies (Table 1 and Figure 2). The first one was identified in the Old Order Amish in Lancaster County, Pennsylvania. Known as the “Chicken Breast Disease” in the Amish community, this “Amish Nemaline Myopathy” (ANM) is a severe myopathy disease with infantile lethality (Johnston et al., 2000). ANM infants exhibit tremors and muscle weakness, followed by the development of contractures and progressive chest deformation due to weakness of the respiratory muscles. Death from respiratory insufficiency usually occurs in the second year. ANM has an incidence of 1 in ~500 births in the Amish communities in Pennsylvania and Ohio (Johnston et al., 2000). No effective treatment is currently available.

**Table 1:** *****TNNT1*** myopathies**.

**Ethnicity**	**Mutation**	**Phenotype**	**References**
Old order amish	E180ter	Recessive NM	Johnston et al., 2000; Jin et al., 2003
Dutch	ΔExon 8	Recessive NM	van der Pol et al., 2014
Dutch	ΔExon 14	Recessive NM	van der Pol et al., 2014
Hispanic, New York	S108ter	Recessive NM	Marra et al., 2015
Palestinian	L203ter	Recessive NM	Abdulhaq et al., 2016

*NM, Nemaline Myopathy*.

The genetic causes of ANM is a nonsense mutation in exon 11 of *TNNT1* gene converting the codon Glu^180^ to a premature stop codon to truncate the slow TnT polypeptide chain by deleting the C-terminal 83 amino acids (Johnston et al., 2000). The deletion of the C-terminal segment of slow skeletal muscle TnT by the E180ter mutation causes a loss of the binding sites for TnI, TnC and the tropomyosin-binding site 2 in the T2 region (Jin et al., 2003; Figure 2). Although, the middle region tropomyosin binding site 1 remains intact, the truncated slow TnT is not able to form troponin complex or incorporate into the myofilament (Wang et al., 2005). This phenotype demonstrates the necessity of the two-site anchoring of troponin on the thin filament in the assembly and function of the thin filament regulatory system (Jin and Chong, 2010).

The truncated ANM slow TnT fragment is not detectable in the patient muscle (Jin et al., 2003), indicating a rapid degradation of non-myofilament associated TnT protein and fragments in muscle cells (Wang et al., 2005). This effective removal of mutant or damaged TnT from the myocytes when they are not integrated in the myofibrils is an important protective mechanism to avoid cytotoxic effect (Jeong et al., 2009). This mechanism also explains how the various *TNNT1* mutations reported to date all present as recessively inherited diseases (Johnston et al., 2000; van der Pol et al., 2014; Marra et al., 2015; Abdulhaq et al., 2016). On the other hand, this mechanism converts a potentially dominant negative mutation into a recessive mutation, which calls for more extensive genetic screening of TnT mutations in the clinical diagnosis of recessive myopathies.

Based on the structural and functional defect of ANM slow TnT mutant, the molecular basis of the pathogenesis and pathophysiology of ANM is the complete loss of slow TnT protein in slow muscle fibers (Jin et al., 2003; Wang et al., 2005). The loss of slow TnT causes atrophy and degeneration of slow twitch muscle fibers that are essential for many vital physiological activities (Jin et al., 2003). In a transgenic mouse models of ANM, slow TnT deficiency caused significant decreases in the contents of type I slow fibers in diaphragm and soleus muscles (Feng et al., 2009; Wei et al., 2014). Although, the slow TnT deficient slow fibers had active regeneration and hypertrophic growth of type II fast fibers, the muscles showed significantly decreased fatigue resistance (Feng et al., 2009; Wei et al., 2014), consistent with the pathophysiological phenotype of posture muscle weakness and respiratory muscle failure in ANM patients (Johnston et al., 2000).

The identification of ANM and subsequent mechanistic studies have promoted clinical awareness of *TNNT1* myopathy and its testing in the clinical diagnosis of myopathies. As results, several recent reports have identified four more *TNNT1* mutations in non-Amish ethnic groups, which cause nemaline myopathies clinically similar to ANM (Table 1). *TNNT1* myopathies are, therefore, no longer considered as an isolated disease of the Amish, but are of increasing medical importance. The increasing application of genetic screening is anticipated to identify more *TNNT1* myopathy mutations.

A nonsense mutation in the exon 9 of *TNNT1* gene at codon Ser^108^ was found in a Hispanic patient in New York City with clinical and histological features were very much like that of ANM, including severe respiratory muscle weakness, type I fiber atrophy and compensatory hypertrophy of type II fibers (Marra et al., 2015). The *TNNT1* S108ter mutation is predicted to result in a truncated slow TnT protein missing the C-terminal 155 amino acids. Therefore, the similar recessive phenotypes of the ANM E180ter and S108ter mutations are based on their loss of the T2 region TnI and TnC binding sites as well as the tropomyosin-binding site 2 (Figures 1, 2; Jin and Chong, 2010). Recent biochemical characterization further demonstrated that the Ser^108^ truncation of slow TnT also partially damages the middle region tropomyosin binding site 1 (Amarasinghe et al., 2016), which makes it more unlikely to incorporate into the myofibrils.

A genomic DNA rearrangement in *TNNT1* gene (c.574_577 delins TAGTGCTGT) was reported in 9 Palestinian patients from 7 unrelated families with recessively inherited NM (Abdulhaq et al., 2016). This mutation leads to aberrant splicing to truncate the slow TnT polypeptide at Leu^203^ (Figures 1, 2). The patients presented with recessive NM phenotypes very similar to that of ANM (Abdulhaq et al., 2016). Biochemical studies demonstrated that although slow TnT truncated at Leu^203^ retains both tropomyosin-binding sites 1 and 2, the inability of forming troponin complex due to the loss of TnI and TnC binding sites decreased the binding affinity for tropomyosin, especially at high calcium (Amarasinghe et al., 2016). This loss of function and the loss of the highly conserved C-terminal segment may be responsible for the recessive myopathy phenotype of the Leu^203^ truncation.

Another case report of a Dutch patient of inherited nemaline myopathy described two new *TNNT1* NM mutations (van der Pol et al., 2014). The patient also presented with phenotypes of severe slow skeletal muscle atrophy and weakness similar to that of ANM. Molecular diagnosis identified that the patient is a compound heterozygote of a mutation in intron 8 of the *TNNT1* gene that causes aberrant exclusion of exon 8-encoded sequence and another mutation that causes exclusion of the exon 14-encoded segment (Figure 2) (van der Pol et al., 2014). The deletion of exon 8 segment partially destroys the T1 region tropomyosin-binding site 1 (Figure 1) but preserves the high-affinity binding site 2 (Jin and Chong, 2010), whereas deletion of the exon 14-encoded C-terminal end segment would not directly affect either of the tropomyosin-binding sites, nor the binding sites for TnI and TnC.

A recent study found that slow TnT with the internal deletion of the exon 8-encoded segment has drastically decreased binding affinity for tropomyosin, which is much lower than that of tropomyosin binding site 2 alone. Deletion of the N-terminal variable region partially restored the binding affinity of exon 8-deleted slow TnT (Amarasinghe et al., 2016). These observations indicate that deletion of the exon 8-encoded segment not only directly damages the middle region tropomyosin-binding site 1 but also augments the effect of the N-terminal region on reducing the binding affinity of site 2. Therefore, the N-terminal variable region provides a potential target for the treatment of the myopathy caused by slow TnT exon 8 deletion.

The molecular mechanism for slow TnT exon 14 truncation to cause recessive myopathy remains to be investigated. As described in Section Structure-Function Relationship of Troponin T, there are evidence that the C-terminal end segment of TnT may contribute to the inhibitory regulation of myofilament ATPase (Morimoto et al., 1999; Mukherjea et al., 1999; Franklin et al., 2012). More investigation along this line would help to understand the pathogenic mechanism of this aberrant splicing mutation of *TNNT1* gene.

## Perspectives: what have been learned from the pathogenic mutations of slow skeletal muscle TnT

Through isoform gene regulation, alternative RNA splicing and posttranslational modifications, structural and functional variations of TnT modulate striated muscle contractility. The fact that the loss of only the slow isoform of TnT causes lethal myopathy regardless of the mixed composition and expression of slow and fast TnTs in human skeletal muscles demonstrates the functional divergence and necessity of the fiber type-specific TnT isoforms. The critical role of slow skeletal muscle fibers and the structural-function relationship of slow muscle TnT demonstrated by the lethal myopathic mutations and mechanistic studies summarized in this review provide many novel insights into the structure-function relationship of TnT and troponin regulation of striated muscle contraction.

An intriguing feature of the 5 myopathic *TNNT1* mutations reported to date is that they all presented as recessively inherited diseases (Johnston et al., 2000; van der Pol et al., 2014; Marra et al., 2015; Abdulhaq et al., 2016). To fully understand the molecular basis of these different structural defects of slow TnT for causing complete loss of function from inability of incorporating into the myofilament will help to further understand the structure-function relationship of TnT and troponin regulation of muscle contraction.

Genetically modified mice with a knockdown of the expression of slow TnT exhibited decreased muscle resistance to fatigue (Feng et al., 2009). However, carriers of ANM (and other *TNNT1* NM) did not report notable clinical symptom (Johnston et al., 1997). Therefore, it would be worth further investigating whether the haploid insufficiency in carriers of these recessive *TNNT1* myopathies may cause conditional slow TnT deficiency with symptoms such as experiencing conditional fatigue intolerance and other slow muscle-related dysfunctions.

The potential cytotoxicity of non-myofilament associated slow TnT fragments (Jeong et al., 2009) in the muscle cells of both homozygote patients and heterozygote carriers is also worth investigating. Exhaustive work load or muscle wasting conditions may produce peaks of myofilament decay to add to the existing pool of the mutant slow TnT fragment, which may overwhelm the capacity of protein turnover system in muscle cells to cause apoptosis and inflammatory damage, although such dominant negative phenotype might only be a transient state in the muscle of carriers.

The transient expression of cardiac TnT and embryonic splice forms of fast skeletal muscle TnT in embryonic and neonatal skeletal muscle may explain the postnatal onset of ANM (Jin et al., 2003). This observation suggests a potential compensation of the fetal forms of TnT for the loss of slow TnT in ANM neonatal skeletal muscles, which may be explored as a therapeutic target. This approach would require an activation of cardiac TnT expression or embryonic alternative splicing pathways of fast TnT in adult slow skeletal muscle fibers.

Soleus muscle of slow TnT knockout mouse maintains a slow fiber cellular environment and exhibits signs of active regeneration (Wei et al., 2014). This is a plausible observation suggesting that a restoration of slow TnT in slow muscle fibers of *TNNT1* myopathy patients should be able to readily rescue muscle growth and functions. The maintained slow fiber cellular environment and active regeneration also indicate that translational read-through of the nonsense stop codon in the muscles of ANM and S108ter patients may effectively restore muscle function and growth.

Since *TNNT1* myopathies is no longer considered as a isolated disease of the Amish, and the power of genetic testing is anticipated to identify more myopathic mutations of the gene, researchers are urged to add joint effort in TnT gene expression and structural-function relationship studies, including the utilization of genetically modified mouse models of the human diseases, toward the development of effective targeted treatment of these lethal muscle diseases.

In conclusion, *TNNT1* myopathies demonstrate an excellent example for what we can learn from pathogenic mutations of a myofilament protein as well as how knowledges learned from protein structure-function relationship research can help us to understand the pathogenesis and pathophysiology of genetic diseases. Therefore, we hope this focused review will benefit readers with the vision beyond *TNNT1* myopathy studies.

## Author contributions

AM: Drafting and revising the text, making figures, approval submission; JJ: Deciding the topic and contents, drafting and revising the text, making figures, approval submission.

### Conflict of interest statement

The authors declare that the research was conducted in the absence of any commercial or financial relationships that could be construed as a potential conflict of interest.
